# Genetic mutation profiles and immune microenvironment analysis of pulmonary enteric adenocarcinoma

**DOI:** 10.1186/s13000-022-01206-7

**Published:** 2022-02-16

**Authors:** Min Xie, Dong Chen, Yong Li, Xiansheng Liu, Dong Kuang, Xiaochen Li

**Affiliations:** 1 Department of Pulmonary and Critical Care Medicine, Tongji Hospital, Tongji Medical College, Huazhong University of Science and Technology, 1095 Jiefang Avenue, Wuhan, 430030 China; 2 Key Laboratory of Respiratory Diseases, National Ministry of Health of the People’s Republic of China and National Clinical Research Center for Respiratory Disease, 1095 Jiefang Avenue, Wuhan, 430030 China; 3 Department of Pathology, Tongji Hospital, Tongji Medical College, Huazhong University of Science and Technology, Wuhan, China

**Keywords:** Pulmonary enteric adenocarcinoma, PD-L1, Genetic mutation, Immune microenvironment

## Abstract

**Background:**

Pulmonary enteric adenocarcinoma (PEAC) has distinctive clinical outcomes, radiographic, pathological and molecular characteristics. The prognosis of patients with PEAC was poor. However, molecular profiles and therapeutic biomarkers of PEAC remain elusive.

**Methods:**

In the present study, the hospitalized patients with PEAC admitted to Tongji Hospital in Wuhan from January 1, 2014 to November 20, 2020 were retrospectively enrolled and followed until December 10, 2020. Comprehensive genomic profiling of tumor tissue from the PEAC patients were performed and compared with lung adenocarcinoma, colorectal cancer and metastatic colorectal carcinoma. Tumor immune microenvironment analysis were evaluated.

**Results:**

There were 10 patients with PEAC enrolled. 70% of patients were male and the median age of onset was 63 years (interquartile range, 55–72). There were six early-stage patients (Stage IA to IIB) and four stage IV patients. Molecular analysis revealed the most common gene mutations included TP53 (57%, 4/7) and KRAS (57%, 4/7) mutations. There were 40% mutations occurred in genes encoding receptor tyrosine kinases (RTKs). 100% of patients (8/8) were microsatellite stability (MSS). The median level of TMB was 6.0 (interquartile range, 4.5–7.0) mutations/Mb. Three of 10 patients showed low PD-L1 expression (tumor proportion score < 10%) and the others were PD-L1 negative. A small subset of CD8+, CD3+, CD68+ T cells were observed and were mainly distributed in the cancer stroma.

**Conclusion:**

This study demonstrated that PEAC was characterized by low-frequency RTK gene mutation, high KRAS mutation, low PD-L1 expression, low TMB, and low CD8+ T cells infiltration.

**Supplementary Information:**

The online version contains supplementary material available at 10.1186/s13000-022-01206-7.

## Background

Pulmonary enteric adenocarcinoma (PEAC) was classified as a rare variant of invasive adenocarcinoma in 2011 [1] and included in the World Health Organization classification of lung tumors until 2015 [2]. PEAC and pulmonary invasive mucinous adenocarcinoma showed common histomorphological characteristics featured by columnar cells with mucin production. Furthermore, PEAC are similar to metastatic colorectal carcinoma (MCC) in histomorphologic and immunohistochemical features with more than 50% enteric pattern. It is currently difficult to identify PEAC based on information from conventional morphology, immunohistochemistry and radiology. It is necessary to clinically exclude metastases from colorectal carcinoma before the final diagnosis. Investigations on molecular profiles of PEAC indicated that it could be used as potential optimal markers for differential diagnosis [3–5].

The treatment of lung adenocarcinoma has been improved in the past two decades with increased understanding in molecular genetics. Targeted therapies against driver mutations have been a major breakthrough in treatment of non-small cell lung cancer (NSCLC). The development of immune checkpoint inhibitor (ICI) represents another promising therapy for advanced NSCLC [6]. A number of biomarkers including programmed cell death ligand 1 (PD-L1) expression, tumor mutational burden (TMB), microsatellite instability (MSI), and CD8+ tumor stroma-infiltrating lymphocyte density have been used to predict response to immunotherapy [7–11].

Patients with PEAC were reported to have a higher rate of lymph node or distant metastases at diagnosis and worse prognosis than other types of lung adenocarcinomas [12, 13]. In view of poor survival after traditional therapy, the understanding of molecular genetics and inhibitory immune checkpoints might provide novel treatment options for PEAC. However, molecular profiles and therapeutic biomarkers of PEAC remain elusive. In the present study, we performed comprehensive genomic profiling and tumor immune microenvironment analysis of PEAC. The molecular profiles were compared with lung adenocarcinoma (LUAD), colorectal cancer (CRC) and MCC.

## Methods

### Patients and samples

This study was a retrospective cohort study of hospitalized patients with PEAC enrolled at Tongji Hospital, Huazhong University of Science and Technology in Wuhan from January 1, 2014 to November 20, 2020. This study was approved by Institutional Review Board of Tongji Hospital, Tongji Medical College, Huazhong University of Science and Technology. The diagnosis of PEAC was based on clinical, radiological and pathological information of each patient. Pathologic slides were evaluated by a pathologist to confirm the diagnosis of PEAC, which was defined as a pulmonary adenocarcinoma with an enteric differentiation component exceeding 50%. The possibility of intestinal cancer metastasis in each patient was excluded according to abdominal CT, FDG-PET, gastroscopy and colonoscopy. Patient data including demographic information, laboratory, radiological, and immunohistochemical examinations during hospitalization were extracted from electronic medical records. Patient outcomes were obtained by telephone interview. The last follow-up date was December 10, 2020.

Seven patient tumor tissue samples were sent to Burning Rock Biotech (Guangzhou, China) laboratory which is a College of American Pathologists-accredited and Clinical Laboratory Improvement Amendments-certified clinical laboratory for genetic profiling. The gene panel used in the present study consisting of 520 cancer-related genes (OncoScreen Plus). Two patient tissue samples were detected for common driver gene mutation. The comparison cohorts included LUAD and CRC patients from TCGA and MCC patients from MSKCC, which are available on the website [14, 15]. The LUAD cohort consists of 566 patients with lung adenocarcinoma. The CRC cohort consists of 594 patients with colorectal adenocarcinoma. The MCC cohort consists of 1099 patients with metastatic colorectal cancer. Patient dataset including clinical and genomic information were obtained from the cBioPoratal for cancer genomics (accessed on 14 August 2020).

### DNA extraction

The QIAamp DNA FFPE Tissue Kit (Qiagen, UK) were used to extract tumor DNA from FFPE tumor samples according to the manufacturer’s instructions. The Qubit dsDNA HS Assay Kit (Life Technologies, Carlsbad, USA) were used to measure DNA concentration.

### Library construction and sequencing

The M220 Focused-ultrasonicator (Covaris, Woburn, MA, USA) was used to shear DNA, followed by end repair, phosphorylation, and adaptor ligation. The Agencourt AMPure XP beads (Beckman Coulter, Brea, CA, USA) were used to select DNA fragments with the range of 200–400 bp. Then, hybridization with capture probe baits, hybrid selection with magnetic beads, and PCR amplification were performed. Target capture was performed with a commercially-available panel of 520 cancer-related genes (OncoScreen Plus). DNA quality and fragment size were assessed by Bioanalyzer 2100 (Agilent, CA, USA). The indexed samples were sequenced on Illumina NextSeq 500 paired-end system (Illumina, Inc., Hayward, CA, USA).

### Sequence data analysis

The paired-end reads were mapped to the human genome (hg19) by Burrows-Wheeler aligner v.0.7.10 [16]. Local alignment optimization, variant calling, and annotation were performed with the Genome Analysis Toolkit v.3.2 [17] and VarScan v.2.4.3 [18]. DNA translocation analysis was performed with Factera v.1.4.3 [19]. The variants were filtered with the VarScan filter pipeline, and loci with depths of less than 100 were filtered out. Germline mutations were also filtered out by sequencing matched white blood cells from the samples. Base-calling in tissue samples required at least eight supporting reads for single nucleotide variations and five supporting reads for insertion-deletion variations, respectively. Variants with population frequencies of over 0.1% on the Exome Aggregation Consortium, 1000 Genomes, dbSNP, and ESP6500SI-V2 databases were grouped as single-nucleotide polymorphisms and excluded from further analysis. The remaining variants were annotated with ANNOVAR (2016-02-01 release) [20] and SnpEff v.3.6 [21].

### Tumor microenvironment analysis

Seven patients were evaluated for the PD-L1 expression and microenvironment in tumor slides with the PANO 7-plex IHC kit, cat 0004100100 (Panovue, Beijing, China). Expression of surface markers associated with the tumor microenvironment including PD-1, PD-L1, CD3, CD8, CD68, CD56 and CD163 were analyzed through multiplex staining and multispectral imaging [22]. Different primary antibodies were sequentially applied, followed by horseradish peroxidase-conjugated secondary antibody incubation and tyramide signal amplification. The slides were microwave heat-treated after each TSA operation. Nuclei were stained with 4′-6′-diamidino-2-phenylindole (DAPI, Sigma-Aldrich) after all the human antigens had been labelled. To obtain multispectral images, the stained slides were scanned using the Mantra System (PerkinElmer, Waltham, Massachusetts, US), which captures the fluorescent spectra at 20-nm wavelength intervals from 420 to 720 nm with identical exposure time; the scans were combined to build a single stack image. Images of unstained and single-stained sections were used to extract the spectrum of autofluorescence of tissues and each fluorescein, respectively. The extracted images were further used to establish a spectral library required for multispectral unmixing by inForm image analysis software (PerkinElmer, Waltham, Massachusetts, US). Using this spectral library, we obtained reconstructed images of sections with the autofluorescence removed. Three patients were evaluated for the PD-L1 expression of tumor with IHC 22C3 pharmaDx (Daka, Glostrup, Denmark). PD-L1 expression is determined using Tumor Proportion Score (TPS), which is the percentage of viable tumor cells showing partial or complete membrane staining at any intensity.

### Microsatellite stability status

FFPE prepared sections were immunostained with automated immunostainer (Dako/Agilent Autostainer Link 48). Primary antibody specific for MLH1 (clone ES05, mouse), PMS2 (clone EP51, rabbit), MSH2 (clone FE11, mouse), and MSH6 (clone EP49, rabbit) (all Readyto-Use, from Dako, Glostrup, Denmark) was applied on the sections according to the manufacturer’s directions. Bound antibody was visualized using the EnVision Kit (Dako, Glostrup, Denmark). Nuclear staining in cancer cells with any intensity was recognized as positive. And nuclear staining in stromal or inflammatory cells was additionally recorded as an internal control. Suspected MMR deficiency was defined as complete loss of at least one of the 4 MMR proteins in all tumor cells.

### Statistical analysis

The continuous variables were presented as mean or median. The categorical variables were presented as frequencies. Unpaired Wilcoxon signed-rank test was used to compare continuous variables, while two-sided Fisher’s exact tests were used to compare categorical variables, as appropriate. *P* < 0.05 was considered statistically significant. All bioinformatics analyses were performed with R (v.3.5.3, the R Foundation for Statistical Computing, Vienna, Austria).

## Results

### Clinical and radiological characteristics

A total of 10 patients with PEAC were enrolled. Histopathological diagnoses of PEAC from tumor tissues (three biopsy samples from three patients P08-P10 and seven surgical resections from seven patients P01-P07) after hematoxylin and eosin staining were confirmed (Supplemental Fig. 1). 70% of patients (7/10) were male and the age at diagnosis ranged from 43 to 76 years with the median age at diagnosis was 63 years (interquartile range, 55–72). A half of patients (5/10) were current smokers with a median smoking history of 30 pack years. The serum levels of carcinoembryonic antigen were elevated in a half of patients. There were seven patients with tumor located in right lower lobes and three of them showed similar radiological features to pneumonic infiltrate or consolidation. There were six early-stage patients (Stage IA to IIB). Four patients showed lymph node and distant metastases (Stage IV), which affected the pleural, breast, pericardium and bone. There were seven patients underwent surgical treatment including one patient with pleural metastasis underwent lobectomy and pleurotomy. The median disease-free survival was 20.5 months (interquartile range, 16–28.3). Two patients with early-stage (IB and IIB) died 13–14 months after diagnosis and one of them died of pulmonary tuberculosis. The clinical and radiological data are summarized in Table 1.

**Table 1:** Demographic, clinical and radiological characteristics of the PEAC patients

Case ID	Sex	Age at diagnosis	Smoking history	Pack years	CEA (ng/ml)	Site	TNM classification	Stage	Surgery	Date of diagnosis	DFS (m)	OS (m)	Vital status
P01	Male	75	Current smoker	30	7.3	LUL	pT2aN0M0	IB	Yes	2014.8.13	MD	14	Dead
P02	Male	43	Current smoker	15	24.5	RLL	pT3N2M1	IV	Yes	2014.1.6	24	NA	Alive
P03	Male	55	Current smoker	20	3.7	RLL	pT1bN0M0	IA	Yes	2019.3.1	17	NA	Alive
P04	Male	70	Nonsmoker	NA	1.9	RLL	pT3N0M0	IIB	Yes	2017.1.5	41	NA	Alive
P05	Male	56	Current smoker	30	6.2	RLL	pT3N0M0	IIB	Yes	2018.12.28	13	13	Dead
P06	Male	61	Nonsmoker	NA	2.1	RUL	pT2aN0M0	IB	Yes	2016.12.23	MD	MD	LTF
P07	Female	64	Nonsmoker	NA	2.5	RLL	pT3N0M0	IIB	Yes	2018.8.30	MD	MD	LTF
P08	Female	53	Nonsmoker	NA	15.6	RLL	cT4N3M1	IV	No	2020.9.15	NA	NA	Alive
P09	Male	72	Current smoker	50	204.5	LUL	cT4N3M1	IV	No	2020.9.16	NA	NA	Alive
P10	Female	76	Nonsmoker	NA	3.4	BL	cT4N2M0	IV	No	2020.11.13	NA	NA	Alive

*LUL* left upper lobe, *RLL* right lower lobe, *RUL* right upper lobe, *LUL* left upper lobe, *BL* bilateral lungs, *MD* missing data, *LTF* lost to follow-up, *NA* not applicable

### Immunohistochemical analysis

Immunohistochemical analysis revealed that patients were 100% (10/10) positive for CK7, 60% (6/10) positive for TTF-1, 63% (5/8) positive for Napsin A; 50% (3/6) positive for CK20, 80% (8/10) positive for CDX-2, 70% (7/10) positive for villin, 78% (7/9) positive for P53, 17% (1/6) positive for SATB, whereas patients were 100% (0/8) negative for ALK fusion (Table 2). For Ki-67, there were eight patients had high (≥10%) expression, and two patients had low expression.

**Table 2:**
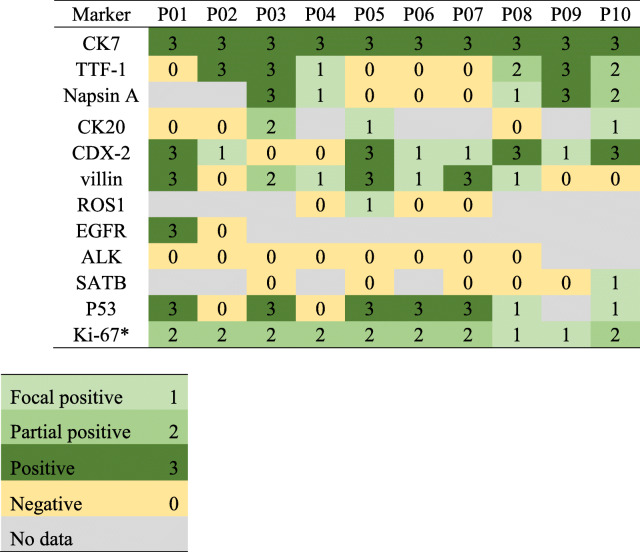
Immunohistochemical features in the patients with PEAC

*Ki-67 index is defined by the percentage of tumor cells with positive nuclear staining out of all tumor cells. 1: < 10%; 2: ≥10%

### Genetic mutation spectrum analysis in the PEAC cohort

**Fig. 1:**
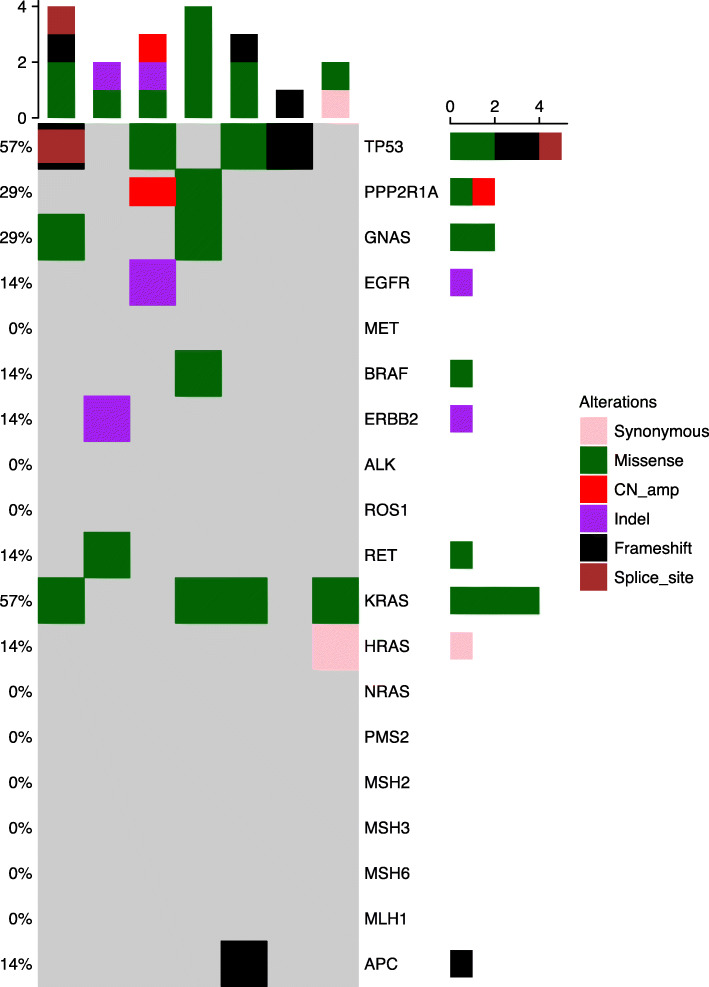
Mutation spectrum analysis heatmap in seven pulmonary enteric adenocarcinoma patients (P01-P06 and P09)

Targeted sequencing of 520 cancer related genes in seven patients (P01-P06 and P9) revealed 69 genomic alterations in 54 genes, including 34 missenses (49%), 9 frameshift (13%), 9 copy number amplifications (13%), 6 stop-gained mutations (9%), 2 splice acceptor variant (3%), 2 splice region variants (3%) and so on (Supplemental Table 1). Each patient carried at least one genetic mutation identified by sequencing. The most common gene mutations included TP53 (57%, 4/7), KRAS (57%, 4/7) mutations (Fig. 1). EGFR (19del) mutation was detected in a stage IA patient (P03, Fig. 1), an ERBB2 (20ins) mutation in a stage IV patient (P02), and a BRAF (V600M) mutation in a stage IIB patient (P04). An APC mutation was detected in a stage IIB patient (P05).

Common driver mutations (EGFR, ALK, BRAF, ERBB2, KRAS, MET, RET, and ROS1) analysis were performed in lung adenocarcinoma tissue samples from two PEAC patients (P08 and P10) using next-generation sequencing. Only KRAS mutation was detected in one patient (P08).

**Fig. 2:**
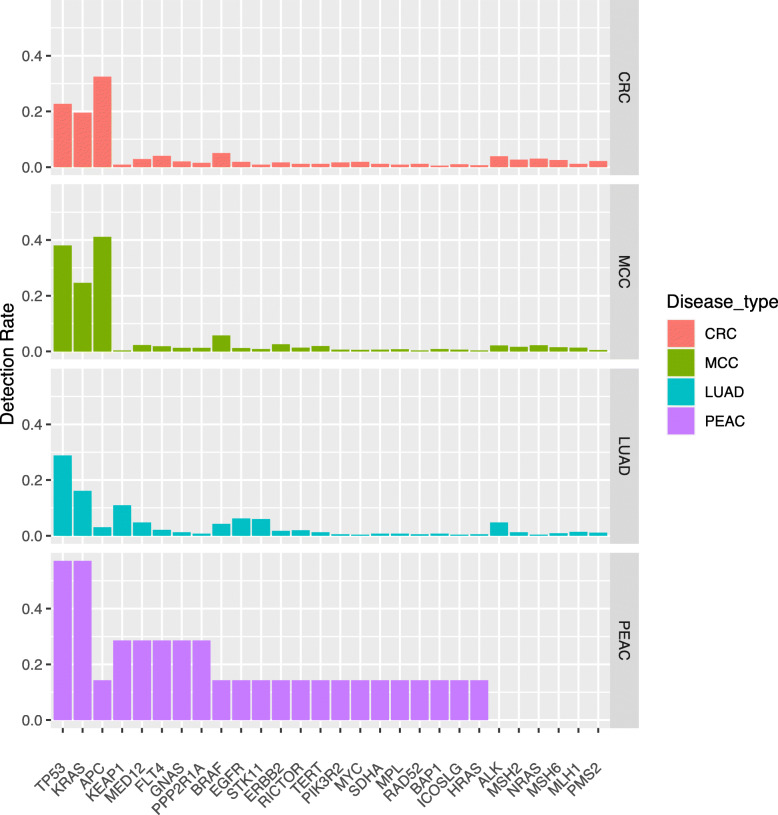
Detection rate statistics in the PEAC cohort and comparison with TCGA and the MSKCC databases. CRC, colorectal cancer; LUAD, lung adenocarcinoma; MCC, metastatic colorectal carcinoma; PEAC, pulmonary enteric adenocarcinoma

In our study of seven PEAC patients (P01-P06 and P09), genetic mutations in 414 of 520 targeted genes were comparable with LUAD, CRC, MCC patients from TCGA and the MSKCC databases. The mutation rates of PPP2R1A, KEAP1, KRAS, MED12, TP53 and GNAS were significantly different between PEAC patients and MCC patients (all *P* < 0.05) (Fig. 2). APC mutation was rare in LUAD but was common in CRC and MCC. Interestingly, mutations in KRAS were more frequently occurred in PEAC than other three tumor types.

**Fig. 3:**
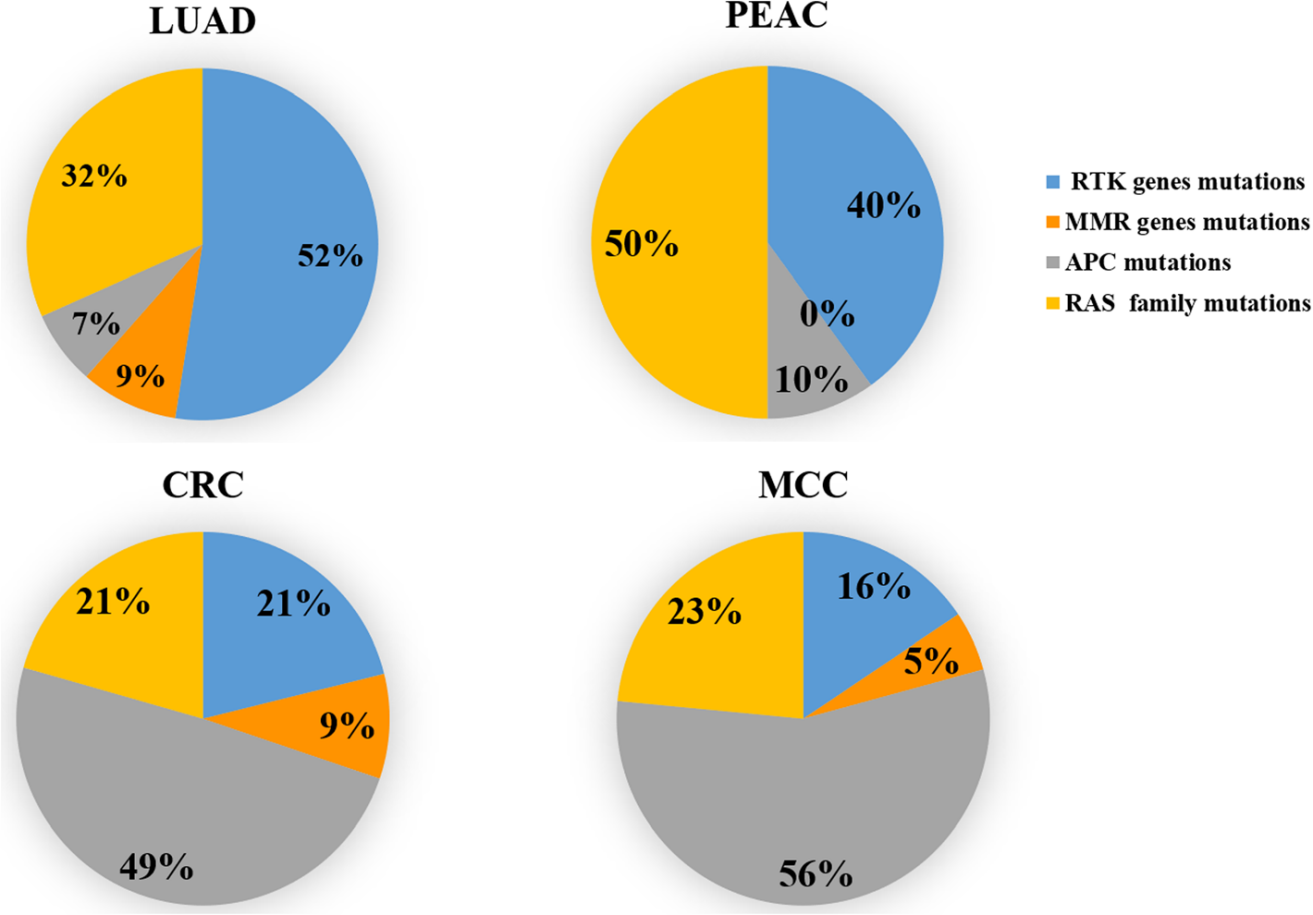
Functional enrichment analysis in the PEAC cohort and comparison with TCGA and the MSKCC databases. CRC, colorectal cancer; LUAD, lung adenocarcinoma; MCC, metastatic colorectal carcinoma; PEAC, pulmonary enteric adenocarcinoma

Functional enrichment analysis based on the panel demonstrated that RAS family mutations account for half of gene mutations in PEAC. There were 40% mutations occurred in genes encoding receptor tyrosine kinases (RTKs) including EGFR, MET, BRAF, ERBB2, ALK, ROS1 and RET (Fig. 3). In general, frequencies of genetic mutation in PEAC patients were similar to those in LUAD patients but were different from those in CRC and MCC patients.

### Single nucleotide variant analysis and mutation signature analysis

**Fig. 4:**
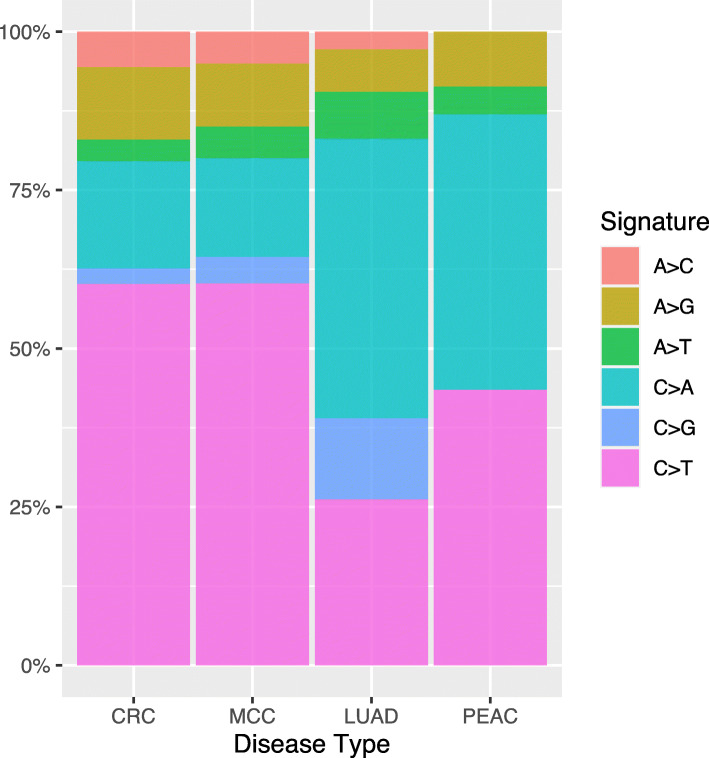
Single nucleotide variant analysis in the PEAC cohort and comparison with TCGA and the MSKCC databases. CRC, colorectal cancer; LUAD, lung adenocarcinoma; MCC, metastatic colorectal carcinoma; PEAC, pulmonary enteric adenocarcinoma

Single nucleotide variant analysis in PEAC showed that the C:G > A:T substitutions were common while the C:G > G:C substitutions were rare (Fig. 4). Comparisons in different tumor types indicated that C > A substitution subtype was more common in PEAC and LUAD, whereas C > T was more frequent in CRC and MCC.

**Fig. 5:**
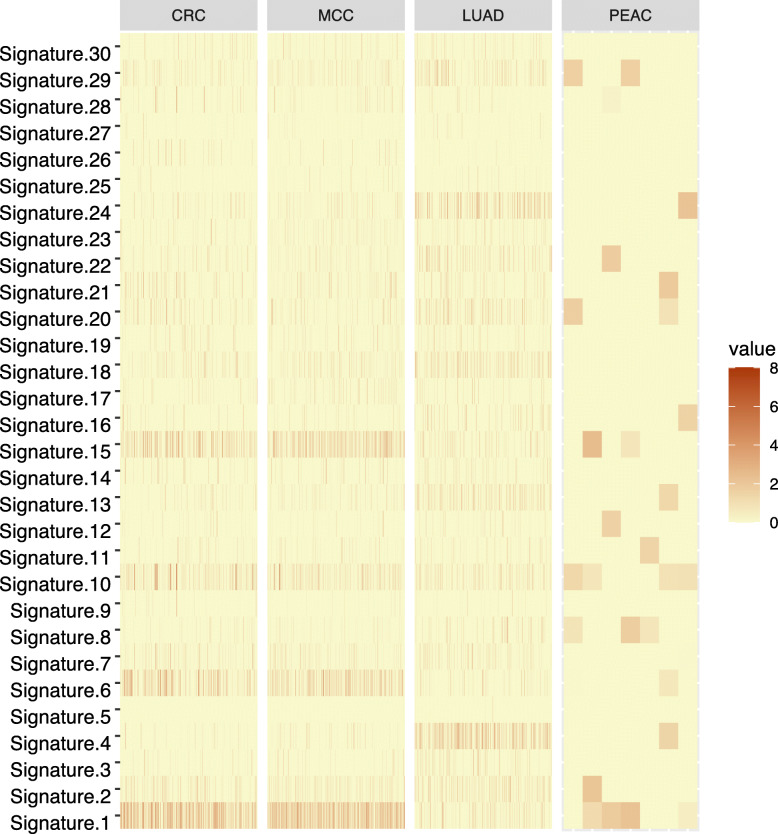
Mutation signature analysis in the PEAC cohort and comparison with TCGA and the MSKCC databases. CRC, colorectal cancer; LUAD, lung adenocarcinoma; MCC, metastatic colorectal carcinoma; PEAC, pulmonary enteric adenocarcinoma

The mutational signature was indicated by the six substitution subtypes (C > A, C > G, C > T, A > T, A > C, and A > G). In general, the profile of mutational signature in PEAC was similar to LUAD (Fig. 5). Signature 1 was presented in all cancer types as the result of an endogenous mutational process initiated by spontaneous deamination of 5-methylcytosine. Signature 4 and signature 29 were associated with tobacco use, however, their profiles in PEAC were distinctly different from LUAD. Signature 6 and signature 15 which were associated with defective DNA mismatch repair and microsatellite instability, were more common in colorectal cancer and rare in PEAC and LUAD.

### Tumor mutation burden and microsatellite stability status

As indicated in Table 3, the median level of TMB in seven PEAC patients was 5.98 (interquartile range, 4.49–6.98) mutations/Mb, ranging from 2.99 to 9.97 mutations/Mb. TMB was comparable between PEAC and lung adenocarcinoma across the TCGA cohort (median: 5.78; range: 0.5–48) [23].

**Table 3:** Tumor mutational burden, microsatellite stability status, and multiplex immunohistochemical predictive biomarkers

Biomarker	P01	P02	P03	P04	P05	P06	P07	P08	P09	P10
TMB (mutations/Mb)	6.98	5.98	3.99	4.99	2.99	9.97	–	–	6.98	–
MSI	MSS	MSS	MSS	MSS	MSS	MSS	MSS	–	MSS	–
Tumor PD-L1 (%)	9.27	11.2	0.3	0.33	0.55	2.19	0	0	3.41	0
Stroma PD-L1 (%)	7.07	20.33	3.99	2.73	3.21	2.14	–	–	0.12	–
Tumor PD-1 (%)	8.08	3.06	0.57	3.71	1.57	3.93	–	–	2.67	–
Stroma PD-1 (%)	7.88	14.3	11.82	11.1	5.63	7.04	–	–	3.67	–
Tumor CD8 (%)	2.42	4.64	2.62	3.75	2.48	4.97	–	–	7.65	–
Stroma CD8 (%)	2.84	11	14.8	7.83	13.51	9.59	–	–	6.77	–
Tumor CD3 (%)	8.58	5.77	5.35	6.85	2.86	11.19	–	–	9.91	–
Stroma CD3 (%)	24.1	23.65	43.44	23.92	17.23	24.9	–	–	6.86	–
Tumor CD68 + CD163- (%)	15.13	6.49	4.31	6.74	14.12	12.2	–	–	–	–
Stroma CD68 + CD163- (%)	12.29	22.61	17.6	11.97	7.4	10.3	–	–	–	–
Tumor CD68 + CD163+ (%)	0.53	0.19	0.54	0.18	0.93	0.7	–	–	–	–
Stroma CD68 + CD163+ (%)	0.62	1.82	1.43	2.63	6.99	1.39	–	–	–	–
Tumor CD56 (%)	3.12	0.22	0.18	4.14	1.11	0.36	–	–	–	–
Stroma CD56 (%)	0.81	0.72	0.28	0.93	0.95	0.32	–	–	–	–

*TMB* tumor mutation burden, *MSI* microsatellite instability, *MSS* microsatellite stability

MSI is the molecular fingerprint of a deficient mismatch repair system. Immunohistochemistry analysis for DNA mismatch repair proteins (MLH1, PMS2, MSH2, MSH6) revealed that eight patients (P01-P07 and P09) were microsatellite stability (MSS) with positive for all microsatellite markers (Table 3).

### Immunophenotypes and microenvironment analysis of PEAC

**Fig. 6:**
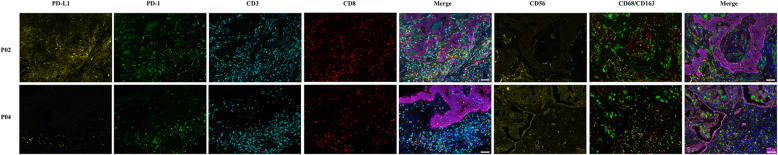
Multiplex immunofluorescence staining for PD-1, PD-L1, CD3, CD8, CD68, CD56 and CD163 in the tumor tissue of the patients with PEAC

Tumor-infiltrating lymphocytes have been associated with response to immunotherapy. Multiplex immunohistochemistry assay was performed in seven patients (P01-P06 and P09) to simultaneously measure the expression of the multiple surface markers associated with the tumor microenvironment, such as PD-1, PD-L1, CD3, CD8, CD68, CD56 and CD163 (Table 3). 57% of the cases (4/7) showed positivity for PD-L1 at low expression level (1–49%). PD-1/PD-L1 expression was higher in cancer stroma compared with cancer nests in most patients. The CD8+, CD3+, CD68+ T cells were mainly distributed in the cancer stroma, whereas the CD56+ T cells were mainly located in cancer nests (Fig. 6).

PD-L1 expressions were evaluated in other three patients (P07, P08 and P10) through immunohistochemical assay using anti-PD-L1 antibody clone 22C3. All three patients showed negative PD-L1 expression (TPS = 0%).

## Discussion

PEAC has distinctive clinical outcomes, radiographic, pathological and molecular characteristics. The prognosis of patients with PEAC was poor [12, 13]. 40% of patients in this study showed lymph node and distant metastases and were diagnosed with stage IV. Two patients with early-stage died 13–14 months after diagnosis.

The radiological study indicated that lesions in PEAC were larger and more solid compared to primary pulmonary invasive adenocarcinoma [24]. In this study, three patients were diagnosed as pneumonic-type of lung cancer. The tumors in seven patients were larger than 5 cm. Nine of the ten patients had unilateral lung carcinomas and seven of them were located in the right lower lobes.

The immunohistochemical markers of lung adenocarcinoma (CK7, TTF-1, Napsin A) and enteric differentiation (CDX-2, CK20, MUC2, villin) were both expressed in PEAC. The combination of CK7+/CDX-2+ was reported to have a high sensitivity (71.3%) and specificity (82%) in differential diagnosis of PEAC from colorectal adenocarcinoma, [25] while combining cadherin-17 (negative) and SATB homeobox 2 (negative) also showed high sensitivity (77.0%) and specificity (100%) [26]. Villin, β-catenin and SATB2 were served as useful immunohistochemical markers for differential diagnosis between PEAC and MCC [27, 28]. Our findings were in line with previous studies and showed positive rates of immunohistochemical markers in PEAC as following: CK7, 100% (10/10); TTF-1, 60% (6/10); Napsin A 63% (5/8); CK20, 50% (3/6); CDX-2, 80% (8/10); villin 70% (7/10).

The profile of driver mutations in PEAC was reported to be similar to NSCLC, but different from MCC [4]. Compared with pulmonary invasive adenocarcinoma, the PEAC indicated a higher incidence (14.3–63%) of KRAS mutation but fewer NSCLC RTK mutations [24, 25, 28]. [5, 29, 30] However, a study revealed that 92.31% (12/13) PEAC patients harbored mutations in well-established RTK genes (EGFR, ALK, ERBB2, BRAF) for NSCLC [4]. Molecular analysis in our study revealed KRAS and TP53 as the most frequently mutated genes (57%, 4/7). Only three cases harbored abnormalities affecting EGFR, ERBB2, and BRAF genes.

As for NSCLC patients without known driver mutation, immunotherapy represents a promising therapeutic strategy for patients with advanced or metastatic disease. Most of ICI targets the PD-1/PD-L1 axis to restore anti-tumor immunity. A number of clinical trials and real-word studies showed that ICI was an important treatment modality for patients with lung adenocarcinoma and significantly improved the prognosis of selected patients [31]. PD-1 targeting agent nivolumab showed treatment effective on NSCLC in the real-world study. Subgroup analyses showed patients with higher PD-L1 expression experienced a greater clinical benefit from nivolumab [32]. PD-L1 expression in tumor cells was identified as a valuable predictor of the efficacy of anti-PD-1/PD-L1 monotherapy in certain NSCLC patients [33]. A previous study in primary lung adenocarcinomas with intestinal differentiation showed that PD-L1 expression was limited to PEAC with a positive rate of 42.9% (3/7), and varied TPS, TPS 20, 50, and 80% respectively [12]. However, in our study four patients showed low PD-L1 expression and the remaining six patients were PD-L1 negative.

Furthermore, MSI, TMB, and CD8+ T-cell tumor-infiltrating lymphocytes have been identified as promising biomarkers to evaluate patients’ survival and response to PD-1/PD-L1 blockade [7–11]. The patients with high MSI were associated with benefit from immunotherapy [34]. Higher TMB also predicted favorable outcome to immunotherapy in NSCLC patients [9]. High MSI and high TMB both reflect instability in tumor cells, and usually occurred simultaneously in same. However, a recent study showed that MSS and TMB-high were more commonly occurred and might be benefit from immunotherapy [8]. The CD8+ T-cell tumor-infiltrating lymphocytes were associated with improved prognosis [10]. A recent study showed that PD-L1 expression on tumor cells in combination with CD8+ T-cell density were predictive biomarkers in patients with inoperable locally advanced NSCLC treated with concurrent chemoradiotherapy. The longest and shortest OS were observed in patients with PD-L1 negative/CD8+ low and PD-L1 positive/CD8+ low tumors respectively [35]. A previous study including 18 Chinese PEAC patients showed that compared with lung adenocarcinomas, PEAC had higher nonsynonymous TMB and MSI [25]. A study of seven Germany PEAC patients revealed a median TMB of 16.8 mutations/Mb which is much higher than that in our study (5.98 mutations/Mb), and none of them were MSI [12]. The mutation load in 13 Chinese PEAC patients in Zhang’s study was similar to our study [4]. The differences in TMB between the previous studies and our studies may be due to the limited sample size, different races, and genetic heterogeneity of PEAC patients. Above all, a majority of tumor in our study were MSS, low TMB, and low CD8+ T cells infiltration.

The analysis of potential genomic biomarkers of immunotherapy in 1000 Chinese patients with cancer showed that lung cancer patients with EGFR mutations had significantly lower TMB than those with wild-type EGFR [36]. The TMB of the patient (P03) with EGFR mutation was 3.99 mutations/Mb in our study. Lung adenocarcinoma with TP53 mutation/STK11-EGFR wild-type was reported to have higher CD8+ T-cell density and PD-L1 expression than other tumor subtypes, and the group of patients had a prolonged progression-free survival from immunotherapy [37]. There were three patients (P01, P05 and P06) harboring TP53 mutation/STK11-EGFR wild-type in our study, nevertheless, no differences were observed in CD8+ T-cell infiltration and PD-L1 expression in the three patients.

The main limitation of this study is the retrospective, observational nature of the study in a single-center with small sample size. It was difficult to draw a comprehensive conclusion regarding molecular genetics profile and predictive biomarkers for immunotherapy in PEAC. Large-scale prospective cohort studies are needed to validate our findings. Additional limit7ations include lacks of treatment response evaluation and analysis of prognostic factors due to the limited cases.

## Conclusions

In summary, this study demonstrated that PEAC was characterized by low-frequency RTK gene mutation, high KRAS mutation, low PD-L1 expression, low TMB, and low CD8+ T cells infiltration, providing important information for the development of therapeutic strategies for patients with PEAC.

## Supplementary Information

**Additional file 1: Supplemental Fig. 1.** Hematoxylin and eosin staining of tumor tissues from all PEAC patients.

**Additional file 2: Supplemental Table 1.** Genetic mutational profile of PEAC using a 520-gene OncoScreen Plus panel.

## Data Availability

All data generated or analyzed during this study are included in this published article and its supplementary information files.

## Abbreviations

PEAC: Pulmonary enteric adenocarcinoma
NSCLC: Non-small cell lung cancer
MC: Metastatic colorectal carcinoma
PD-L1: Programmed cell death ligand 1
TMB: Tumor mutational burden
MSI: Microsatellite instability
LUAD: Lung adenocarcinoma
CRC: Colorectal cancer
RTKs: Encoding receptor tyrosine kinases
MSS: Microsatellite stability
TPS: Tumor Proportion Score
ICI: Immune checkpoint inhibitors
