# Supplementary figures and images for: Genetic mutation profiles and immune microenvironment analysis of pulmonary enteric adenocarcinoma
Source: Diagn Pathol. 2022 Feb 16;17:30. doi: 10.1186/s13000-022-01206-7 (PMC8849039; doi:10.1186/s13000-022-01206-7)

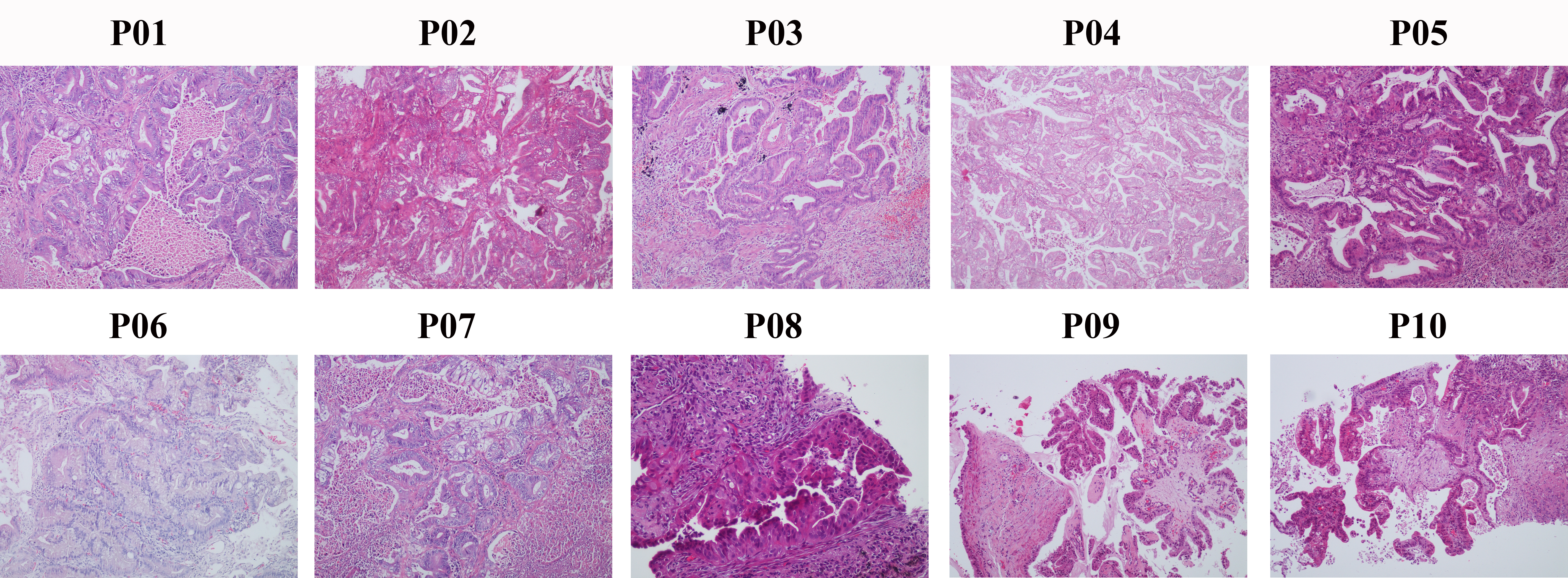

Supplement: Supplementary file 1 — Additional file 1: Supplemental Fig. 1. Hematoxylin and eosin staining of tumor tissues from all PEAC patients. [file 13000_2022_1206_MOESM1_ESM.png]
