# Supplementary material for: Genetic mutation profiles and immune microenvironment analysis of pulmonary enteric adenocarcinoma
Source: Diagn Pathol. 2022 Feb 16;17:30. doi: 10.1186/s13000-022-01206-7 (PMC8849039; doi:10.1186/s13000-022-01206-7)
Supplement: Supplementary file 2 — Additional file 2: Supplemental Table 1. Genetic mutational profile of PEAC using a 520-gene OncoScreen Plus panel. [file 13000_2022_1206_MOESM2_ESM.docx]

Table 1. Genetic mutational profile of PEAC using a 520-gene OncoScreen Plus panel

| Name | Gene | Description | AF | DP | CHR | POS | REF | ALT | VT | Mutation type | EN | HGVS | TranscriptID | |
| --- | --- | --- | --- | --- | --- | --- | --- | --- | --- | --- | --- | --- | --- | --- |
| P01 | KRAS | p.G12V | 12.94% | 3446 | 12 | 25398284 | C | A | SNV | missense_variant | 2 | NM_033360.3(KRAS):c.35G>T(p.Gly12Val) | NM_033360.3 | |
| P01 | TOP2A | p.K1370E | 10.31% | 1406 | 17 | 38548344 | T | C | SNV | missense_variant | 32 | NM_001067.3(TOP2A):c.4108A>G(p.Lys1370Glu) | NM_001067.3 | |
| P01 | TOP2A | p.R73G | 15.83% | 1870 | 17 | 38572725 | T | C | SNV | missense_variant | 3 | NM_001067.3(TOP2A):c.217A>G(p.Arg73Gly) | NM_001067.3 | |
| P01 | TP53 | p.P300fs | 12.54% | 5279 | 17 | 7577034 | CTGGGG | C | INDEL | frameshift_variant | 8 | NM_000546.5(TP53):c.899_903del(p.Pro300fs) | NM_000546.5 | |
| P01 | TP53 | p.T125= | 10.09% | 1090 | 17 | 7579312 | C | A | SNV | splice_region_variant | 4 | NM_000546.5(TP53):c.375G>T(p.Thr125=) | NM_000546.5 | |
| P01 | PIK3R2 | p.Q475H | 11.51% | 843 | 19 | 18276978 | G | T | SNV | missense_variant | 12 | NM_005027.3(PIK3R2):c.1425G>T(p.Gln475His) | NM_005027.3 | |
| P01 | GNAS | p.R1016S | 12.26% | 2650 | 20 | 57485816 | C | A | SNV | missense_variant | 13 | NM_080425.3(GNAS):c.3046C>A(p.Arg1016Ser) | NM_080425.3 | |
| P01 | ICOSLG | p.S248N | 3.71% | 3265 | 21 | 45651282 | C | T | SNV | missense_variant | 5 | NM_001283050.1(ICOSLG):c.743G>A(p.Ser248Asn) | NM_001283050.1 | |
| P01 | PIK3CA | p.H1047R | 11.72% | 2893 | 3 | 1.79E+08 | A | G | SNV | missense_variant | 21 | NM_006218.3(PIK3CA):c.3140A>G(p.His1047Arg) | NM_006218.3 | |
| P01 | FLT4 | c.817-6G>A | 15.97% | 958 | 5 | 1.8E+08 | C | T | SNV | splice_region_variant | 7 | NM_182925.4(FLT4):c.817-6G>A | NM_182925.4 | |
| P01 | FANCG | p.A272S | 4.83% | 2713 | 9 | 35076831 | C | A | SNV | missense_variant | 7 | NM_004629.1(FANCG):c.814G>T(p.Ala272Ser) | NM_004629.1 | |
| P02 | RET | p.M868I | 3.44% | 814 | 10 | 43615190 | G | A | SNV | missense_variant | 14 | NM_020975.4(RET):c.2604G>A(p.Met868Ile) | NM_020975.4 | |
| P02 | MGA | p.A2420V | 3.96% | 1616 | 15 | 42052588 | C | T | SNV | missense_variant | 20 | NM_001164273.1(MGA):c.7259C>T(p.Ala2420Val) | NM_001164273.1 | |
| P02 | ERBB2 | p.Y772_A775dup | 17.61% | 2447 | 17 | 37880981 | A | AGCATACGTGATG | INDEL | disruptive_inframe_insertion | 20 | NM_004448.3(ERBB2):c.2313_2324dup(p.Tyr772_Ala775dup) | NM_004448.3 | |
| P02 | ERG | p.M40I | 5.44% | 1269 | 21 | 39817464 | C | T | SNV | missense_variant | 4 | NM_001136154.1(ERG):c.120G>A(p.Met40Ile) | NM_001136154.1 | |
| P02 | EP300 | p.E1672* | 3.96% | 934 | 22 | 41572485 | G | T | SNV | stop_gained | 30 | NM_001429.3(EP300):c.5014G>T(p.Glu1672*) | NM_001429.3 | |
| P02 | EPHA5 | p.R36* | 4.14% | 531 | 4 | 66535355 | G | A | SNV | stop_gained | 1 | NM_001281765.2(EPHA5):c.106C>T(p.Arg36*) | NM_001281765.2 | |
| P02 | PIK3CG | p.A885T | 4.41% | 1631 | 7 | 1.07E+08 | G | A | SNV | missense_variant | 8 | NM_001282426.1(PIK3CG):c.2653G>A(p.Ala885Thr) | NM_001282426.1 | |
| P03 | RAD52 | p.S157G | 6.24% | 1121 | 12 | 1034690 | T | C | SNV | missense_variant | 7 | NM_001297419.1(RAD52):c.469A>G(p.Ser157Gly) | NM_001297419.1 | |
| P03 | TP53 | p.R273H | 45.89% | 3288 | 17 | 7577120 | C | T | SNV | missense_variant | 8 | NM_000546.5(TP53):c.818G>A(p.Arg273His) | NM_000546.5 | |
| P03 | PPP2R1A | cn_amp | 3.4 | NA | 19 | 19q13.41 | 8 | 7 | CNV | cn_amp | NA | cn_amp | NM_014225.5 | |
| P03 | PMS1 | c.316-2A>T | 7.96% | 930 | 2 | 1.91E+08 | A | T | SNV | splice_acceptor_variant | 4 | NM_000534.4(PMS1):c.316-2A>T | NM_000534.4 | |
| P03 | ZNF217 | cn_amp | 3.2 | NA | 20 | 20q13.2 | 2 | 2 | CNV | cn_amp | NA | cn_amp | NM_006526.2 | |
| P03 | RICTOR | cn_amp | 4 | NA | 5 | 5p13.1 | 39 | 37 | CNV | cn_amp | NA | cn_amp | NM_001285439.1 | |
| P03 | IL7R | cn_amp | 3.7 | NA | 5 | 5p13.2 | 7 | 7 | CNV | cn_amp | NA | cn_amp | NM_002185.3 | |
| P03 | SDHA | cn_amp | 3.8 | NA | 5 | 5p15.33 | 15 | 9 | CNV | cn_amp | NA | cn_amp | NM_004168.3 | |
| P03 | TERT | cn_amp | 5.6 | NA | 5 | 5p15.33 | 16 | 15 | CNV | cn_amp | NA | cn_amp | NM_198253.2 | |
| P03 | INHBA | p.V155F | 4.28% | 1541 | 7 | 41730066 | C | A | SNV | missense_variant | 3 | NM_002192.3(INHBA):c.463G>T(p.Val155Phe) | NM_002192.3 | |
| P03 | EGFR | p.E746_A750del | 35.87% | 2997 | 7 | 55242464 | AGGAATTAAGAGAAGC | A | INDEL | disruptive_inframe_deletion | 19 | NM_005228.3(EGFR):c.2235_2249del(p.Glu746_Ala750del) | NM_005228.3 | |
| P03 | HGF | p.K132E | 6.65% | 1534 | 7 | 81386593 | T | C | SNV | missense_variant | 4 | NM_000601.5(HGF):c.394A>G(p.Lys132Glu) | NM_000601.5 | |
| P03 | CSMD3 | cn_amp | 6.6 | NA | 8 | 8q23.3 | 5 | 4 | CNV | cn_amp | NA | cn_amp | NM_198123.1 | |
| P03 | MYC | cn_amp | 3.7 | NA | 8 | 8q24.21 | 3 | 3 | CNV | cn_amp | NA | cn_amp | NM_002467.4 | |
| P04 | KRAS | p.G12V | 4.18% | 3564 | 12 | 25398284 | C | A | SNV | missense_variant | 2 | NM_033360.3(KRAS):c.35G>T(p.Gly12Val) | NM_033360.3 | |
| P04 | PPP2R1A | p.R182W | 4.98% | 2388 | 19 | 52715979 | C | T | SNV | missense_variant | 5 | NM_014225.5(PPP2R1A):c.544C>T(p.Arg182Trp) | NM_014225.5 | |
| P04 | GNAS | p.R844H | 4.15% | 2364 | 20 | 57484421 | G | A | SNV | missense_variant | 8 | NM_080425.3(GNAS):c.2531G>A(p.Arg844His) | NM_080425.3 | |
| P04 | BAP1 | c.122+1G>T | 5.86% | 1742 | 3 | 52443569 | C | A | SNV | splice_donor_variant | 3 | NM_004656.3(BAP1):c.122+1G>T | NM_004656.3 | |
| P04 | TET2 | p.H1150fs | 3.12% | 1667 | 4 | 1.06E+08 | AC | A | INDEL | frameshift_variant | 4 | NM_001127208.2(TET2):c.3448del(p.His1150fs) | NM_001127208.2 | |
| P04 | FBXW7 | p.E39* | 8.38% | 2935 | 4 | 1.53E+08 | C | A | SNV | stop_gained | 2 | NM_033632.3(FBXW7):c.115G>T(p.Glu39*) | NM_033632.3 | |
| P04 | BRAF | p.V600M | 8.54% | 1978 | 7 | 1.4E+08 | C | T | SNV | missense_variant | 15 | NM_004333.4(BRAF):c.1798G>A(p.Val600Met) | NM_004333.4 | |
| P05 | PTEN | p.A333fs | 16.17% | 1163 | 10 | 89720842 | CA | C | INDEL | frameshift_variant | 8 | NM_000314.6(PTEN):c.996del(p.Ala333fs) | NM_000314.6 | |
| P05 | KRAS | p.G12D | 18.92% | 2442 | 12 | 25398284 | C | T | SNV | missense_variant | 2 | NM_033360.3(KRAS):c.35G>A(p.Gly12Asp) | NM_033360.3 | |
| P05 | NKX2.1 | p.Q301fs | 28.78% | 139 | 14 | 36986787 | T | TGTGC | INDEL | frameshift_variant | 3 | NM_001079668.2(NKX2-1):c.901_902insGCAC(p.Gln301fs) | NM_001079668.2 | |
| P05 | TP53 | p.V197M | 18.39% | 3300 | 17 | 7578260 | C | T | SNV | missense_variant | 6 | NM_000546.5(TP53):c.589G>A(p.Val197Met) | NM_000546.5 | |
| P05 | APC | p.T1556fs | 16.12% | 1377 | 5 | 1.12E+08 | G | GA | INDEL | frameshift_variant | 16 | NM_000038.5(APC):c.4666dup(p.Thr1556fs) | NM_000038.5 | |
| P05 | CDH18 | p.V655M | 15.08% | 1956 | 5 | 19473745 | C | T | SNV | missense_variant | 15 | NM_001291956.1(CDH18):c.1963G>A(p.Val655Met) | NM_001291956.1 | |
| P06 | MPL | p.G600V | 49.36% | 1708 | 1 | 43818334 | G | T | SNV | missense_variant | 12 | NM_005373.2(MPL):c.1799G>T(p.Gly600Val) | NM_005373.2 | |
| P06 | MAP2K1 | p.D67N | 1.29% | 1703 | 15 | 66727483 | G | A | SNV | missense_variant | 2 | NM_002755.3(MAP2K1):c.199G>A(p.Asp67Asn) | NM_002755.3 | |
| P06 | TP53 | p.P322fs | 13.18% | 2511 | 17 | 7576880 | TG | T | INDEL | frameshift_variant | 9 | NM_000546.5(TP53):c.965del(p.Pro322fs) | NM_000546.5 | |
| P06 | KEAP1 | p.Q292* | 3.75% | 4829 | 19 | 10602704 | G | A | SNV | stop_gained | 3 | NM_012289.3(KEAP1):c.874C>T(p.Gln292*) | NM_012289.3 | |
| P06 | KEAP1 | p.Y54C | 23.50% | 1383 | 19 | 10610549 | T | C | SNV | missense_variant | 2 | NM_012289.3(KEAP1):c.161A>G(p.Tyr54Cys) | NM_012289.3 | |
| P06 | SMARCA4 | exon26del | 36.47% | 571 | 19 | 11141974 | SV_Type=DEL;Orientation=FR;Gene_L=SMARCA4;Gene_R=SMARCA4 | Breakpoint=19:11141974_19:11144409 | DEL | large_genomic_rearrangement | Intron25_Intron26 | SMARCA4(NA):c.3546+406_3775-35del | NA |  |
| P06 | IRS1 | p.K79E | 46.54% | 2009 | 2 | 2.28E+08 | T | C | SNV | missense_variant | 1 | NM_005544.2(IRS1):c.235A>G(p.Lys79Glu) | NM_005544.2 | |
| P06 | SNCAIP | p.D10Y | 13.83% | 911 | 5 | 1.22E+08 | G | T | SNV | missense_variant | 3 | NM_001308100.1(SNCAIP):c.28G>T(p.Asp10Tyr) | NM_001308100.1 | |
| P06 | HDAC2 | p.R170K | 6.17% | 1345 | 6 | 1.14E+08 | C | T | SNV | missense_variant | 6 | NM_001527.3(HDAC2):c.509G>A(p.Arg170Lys) | NM_001527.3 | |
| P06 | PTCH1 | p.V1042L | 13.69% | 1023 | 9 | 98220339 | C | A | SNV | missense_variant | 18 | NM_000264.3(PTCH1):c.3124G>T(p.Val1042Leu) | NM_000264.3 | |
| P06 | ARAF | c.97-3_97-2del | 32.47% | 388 | X | 47422616 | TAC | T | INDEL | splice_acceptor_variant | 3 | NM_001256196.1(ARAF):c.97-3_97-2del | NM_001256196.1 | |
| P06 | MED12 | p.K200N | 3.82% | 837 | X | 70340867 | G | C | SNV | missense_variant | 5 | NM_005120.2(MED12):c.600G>C(p.Lys200Asn) | NM_005120.2 | |
| P09 | ZNF217 | cn_amp | 320.00% | NA | 20q13.2 | 20q13.2 | 7 | 6 | NULL | cn_amp | NA | NA | NM_006526.2 | |
| P09 | KRAS | p.G12C | 6.88% | 2600 | 1200.00% | 25398285 | C | A | NULL | missense_variant | 2 | NM_033360.3(KRAS):c.34G>T(p.Gly12Cys) | NM_033360.3 | |
| P09 | CBL | p.Q691= | 9.88% | 972 | 1100.00% | 1.19E+08 | A | G | NULL | synonymous_variant | 13 | NM_005188.3(CBL):c.2073A>G(p.Gln691=) | NM_005188.3 | |
| P09 | HRAS | p.A146= | 5.46% | 604 | 11 | 533465 | G | C | NULL | synonymous_variant | 4 | NM_005343.3(HRAS):c.438C>G(p.Ala146=) | NM_005343.3 | |
| P09 | FANCI | p.R879* | 5.91% | 1929 | 15 | 89838324 | C | T | SNV | stop_gained | 24 | NM_001113378.1(FANCI):c.2635C>T(p.Arg879*) | NM_001113378.1 | |
| P09 | GRIN2A | p.E517* | 15.85% | 1281 | 16 | 9934606 | C | A | SNV | stop_gained | 8 | NM_000833.4(GRIN2A):c.1549G>T(p.Glu517*) | NM_000833.4 | |
| P09 | STK11 | p.M51fs | 11.75% | 502 | 19 | 1207064 | T | TC | INDEL | frameshift_variant | 1 | NM_000455.4(STK11):c.152_153insC(p.Met51fs) | NM_000455.4 | |
| P09 | TIPARP | p.S144L | 6.87% | 2941 | 3 | 1.56E+08 | C | T | SNV | missense_variant | 2 | NM_001184717.1(TIPARP):c.431C>T(p.Ser144Leu) | NM_001184717.1 | |
| P09 | SETD2 | p.S2470fs | 9.49% | 1360 | 3 | 47061264 | CTTCTTTGCTT | C | INDEL | frameshift_variant | 19 | NM_014159.6(SETD2):c.7407_7416del(p.Ser2470fs) | NM_014159.6 | |
| P09 | FLT4 | c.2543-5G>A | 8.29% | 398 | 5 | 1.8E+08 | C | T | SNV | intron_variant | 18 | NM_182925.4(FLT4):c.2543-5G>A | NM_182925.4 | |
| P09 | MED12 | p.G1810C | 17.96% | 540 | X | 70356756 | G | T | SNV | missense_variant | 38 | NM_005120.2(MED12):c.5428G>T(p.Gly1810Cys) | NM_005120.2 | |
| P09 | KEAP1 | p.F478fs | 6.25% | 1136 | 19 | 10600424 | G | GC | NULL | frameshift_variant | 4 | NM_012289.3(KEAP1):c.1430dup(p.Phe478fs) | NM_012289.3 | |

VT, variable type; EN, exon number.
